# Supplementary material for: Metagenomic identification of active methanogens and methanotrophs in serpentinite springs of the Voltri Massif, Italy
Source: PeerJ. 2017 Jan 26;5:e2945. doi: 10.7717/peerj.2945 (PMC5274519; doi:10.7717/peerj.2945)
Supplement: File S6 [file peerj-05-2945-s006.zip › Supp-File6-metagenome-phylosift-taxonomy-krona-graphs/BR2-river-2012a-metagenome-phylosift-taxonomy.html]

Javascript must be enabled to view this page.

abundanceBR2backA1.forward.decontam.derep.adapt\_trim.qual\_trim.fastq.gz98034.269709703798009.322661307886561.58002115854916.067120112931239.012127962573131.987509909362238.5963686681336716.46177024088965.184228790521298.098544126942053.875337361181026.225362284721185.022814737551161.4114042388299.263593688941517.069941294661178.441638893721141.215288219238832.1677212348311.465887445081979.903905286983247.05215109222400.06632447261859.086158911279826.903623903985399.490011755172530.747081777091683.805226283311151.655188299011312.065873339351240.914100798082960.758499274082684.935422071961520.020279055891375.905216742566816.082530296926307.110129572721018.005578627431003.791479552711956.358718345591651.798084927071104.26868363551537.950664655931505.620316370961599.901925758151559.312908281351518.7238908045610301.9724127453131.346145200781517.478647923451699.507455551561132.860926480191424.843552728791339.492016958331349.404698705076382.542499258061954.499742636861581.6347500041208.769757371134068.743439494651238.058678896011179.664518063541179.664518063541063.346802323391259.104665986323523.06829268262688.397384912472492.089976405391834.346296859422407.695506748911168.632776874271318.914405251287.79761568311256.68082611621225.56403654931269.649322878765943.655527739481056.67939470855986.9214210452861827.613005403215503.968499575631815.250226689291546.147907299931133.93352659381062.84617755177

  
