# Supplementary material for: Metagenomic identification of active methanogens and methanotrophs in serpentinite springs of the Voltri Massif, Italy
Source: PeerJ. 2017 Jan 26;5:e2945. doi: 10.7717/peerj.2945 (PMC5274519; doi:10.7717/peerj.2945)
Supplement: File S6 [file peerj-05-2945-s006.zip › Supp-File6-metagenome-phylosift-taxonomy-krona-graphs/BR2-river-2013-metagenome-phylosift-taxonomy.html]

Javascript must be enabled to view this page.

abundanceBR2upA1\_2013.forward.decontam.derep.adapt\_trim.qual\_trim.fastq.gz118636.268440925118264.187138601109751.791726276546.815735806262751.338402740461634.217722277323190.006010027392374.5639273571152452.451239346620789.45272703111894.365866158014455.639647693854339.611195300633685.753864843861551.031799426321801.76435332034583.866473496513184.976918119112822.72637235791855.937828670761385.225007569111253.482034664023503.473048776291854.035964440071472.854903305075246.788673339734716.8555617755612543.26184022767469.516233322424702.884942097461416.028123498281302.488874789724750.092532458894267.939941786222403.732551825032273.598799221259619.790961018319210.142208294641545.678144469971527.283859892861232.321456689582896.173686818522596.186530109751696.511085842492876.922003023142844.369038254941960.322737414161332.360533355891295.325240887081258.2899484182714012.33026644251549.904421483435958.729500251942669.944682186741595.173594432132024.162542044941349.441694696631543.897917672151424.264006661521514.352404516654410.456586091442254.017357219571808.391102125141362.76484703071932.832912050715420.993547029524954.702450777964828.551014485963511.697634858261919.540104989095469.970446148131511.787808764573041.234029621011221.04985738635

  
