# Supplementary material for: Metagenomic identification of active methanogens and methanotrophs in serpentinite springs of the Voltri Massif, Italy
Source: PeerJ. 2017 Jan 26;5:e2945. doi: 10.7717/peerj.2945 (PMC5274519; doi:10.7717/peerj.2945)
Supplement: File S6 [file peerj-05-2945-s006.zip › Supp-File6-metagenome-phylosift-taxonomy-krona-graphs/BR2-spring-2012-metagenome-phylosift-taxonomy.html]

Javascript must be enabled to view this page.

abundanceBR2.forward.decontam.derep.adapt\_trim.qual\_trim.fastq.gz187284.745828128187282.125191978172238.56492630315055.27914316771909.9501132298412120.08692807876834.210489093843649.2465548203665820.64887868163238.636875390322667.22650030248820.9743842548179.458038493238307.912104788821997.000967983416875.02185857848937.59600959312093.71339333992228.424944232291895.371993544272208.575359939849776.021621314583194.962643296112036.211911386124525.524196496864504.727627243593172.792752458622886.471567843883710.99858203452143.451069540193571.784328998133399.785128490353227.7859279826136098.054997665212007.46215680856311.363338724431924.649318286694502.511369884243001.674246589492702.245532218712524.006324415146364.394600816152625.831187268263129.68316663212683.704747279452237.72632792682814.730025232212799.121314629421894.730136646635277.847778436353828.247948380386559.496524964936417.569130222196276.151889157076134.734648091964088.778679685662725.795682185232289.814771081110219.019398723278.001466015044824.540866954942910.420424317231994.338354129841964.715412554711921.18581389635

  
