# Supplementary material for: Metagenomic identification of active methanogens and methanotrophs in serpentinite springs of the Voltri Massif, Italy
Source: PeerJ. 2017 Jan 26;5:e2945. doi: 10.7717/peerj.2945 (PMC5274519; doi:10.7717/peerj.2945)
Supplement: File S6 [file peerj-05-2945-s006.zip › Supp-File6-metagenome-phylosift-taxonomy-krona-graphs/BR2-spring-2013-metagenome-phylosift-taxonomy.html]

Javascript must be enabled to view this page.

abundanceBR2\_2013.forward.decontam.derep.adapt\_trim.qual\_trim.fastq.gz77991.669022757777984.288945369372187.05556553635778.68472126469842.4947862997414525.744855737652783.529684362221183.1607589537134406.06347872593008.32556425687572.432711312671560.87783187001878.52933330121877.670448279188631.350856639168399.796162379025300.437123317412999.138826557992316.449741167661246.9655334864214287.16398905139015.24057340444859.130140803971088.10432107842879.191821521371961.236867495211548.558514647191490.507776094871831.571809278691686.627774443731236.5676067764824.3784045175911842.032329363621705.66479764472878.196035840925840.1796252515121734.243197256141189.437339145081359.642868732611290.967643724251222.2924187158911791.76789229583837.691390556462062.292333908151531.436041159061020.957360772711012.91327855606952.1005063234452524.25384900231029.087090832921060.62845536691913.3856421829891075.016555671951049.934924981391754.516336931321219.791398563631541.365496547461505.558796939771469.752097332081433.94539772438862.1508587842793883.322692071981187.994906225861913.910687761061161.04485773451

  
