# Supplementary material for: Metagenomic identification of active methanogens and methanotrophs in serpentinite springs of the Voltri Massif, Italy
Source: PeerJ. 2017 Jan 26;5:e2945. doi: 10.7717/peerj.2945 (PMC5274519; doi:10.7717/peerj.2945)
Supplement: File S6 [file peerj-05-2945-s006.zip › Supp-File6-metagenome-phylosift-taxonomy-krona-graphs/ESOM-Bin1-Methanobacteriaceae-phylosift-taxonomy.html]

Javascript must be enabled to view this page.

abundancemerged2-merged-mapped-plus-mates-forward.fastq5727.604324176955727.604324176955658.539370471595485.767430270215246.830588936685166.069896002375048.718112157732520.14038318662385.621637989011336.20985938186562.702084681903255.079885076137161.961738930271107.97449262018178.10768289095

  
