# Supplementary material for: Metagenomic identification of active methanogens and methanotrophs in serpentinite springs of the Voltri Massif, Italy
Source: PeerJ. 2017 Jan 26;5:e2945. doi: 10.7717/peerj.2945 (PMC5274519; doi:10.7717/peerj.2945)
Supplement: File S6 [file peerj-05-2945-s006.zip › Supp-File6-metagenome-phylosift-taxonomy-krona-graphs/ESOM-Bin2-Comamonadaceae-Xanthomonadaceae-phylosift-taxonomy.html]

Javascript must be enabled to view this page.

abundancebrown-bins-merged8-merged-mapped-plus-mates-forward.fastq34152.381993336234151.85145206933856.813272850133028.17524909089859.205533640699269.022180636019241.94900071094747.889755036216509.127128075195468.16876866791427.210409260625842.433916212199561.622610808134759.164619936874501.483860068901474.16593320181623083.317893707222383.8829481582630.351662104303519.678980072382495.41728392855714616.6098231133776.1816382394541489.85085413336562.653449348604771.82130985649514.5475399043271199.98841471743398.01433343467803.237636511678535.4917576744532592.639249391071296.319624695546363.590592335664793.58134586182497.5903628346242150.063022185431075.03151109271461.743709444163674.13000116938674.13000116938670.924297872648670.924297872648669.159627502802666.554781617769666.554781617769

  
