# Supplementary material for: Metagenomic identification of active methanogens and methanotrophs in serpentinite springs of the Voltri Massif, Italy
Source: PeerJ. 2017 Jan 26;5:e2945. doi: 10.7717/peerj.2945 (PMC5274519; doi:10.7717/peerj.2945)
Supplement: File S6 [file peerj-05-2945-s006.zip › Supp-File6-metagenome-phylosift-taxonomy-krona-graphs/ESOM-Bin3-Bacteroidetes-phylosift-taxonomy.html]

Javascript must be enabled to view this page.

abundancemerged-blue-bin3-merged-mapped-plus-mates-forward.fastq2310.247475425582310.247475425582286.8727893008341.483111747981328.636290319852428.626435356905824.282088696874224.282088696874224.28208869687422138.460477295632130.76862849788184.260035569282180.36187014072746.3264706071483115.824460339017574.979442896424421.939353488387163.57250044011460.174924199781437.337102451639657.088771405148633.924905896368824.452047872844942.9360585553539461.585576371527277.982697337711138.24400662456428.642745691327824.726607602319243.532948918184436.878364140958524.5855760939724258.632739745045236.15855163268466.712637792249633.704204361532974.555369682614872.416356045458823.202905544348823.2029055443488

  
