# Supplementary material for: Metagenomic identification of active methanogens and methanotrophs in serpentinite springs of the Voltri Massif, Italy
Source: PeerJ. 2017 Jan 26;5:e2945. doi: 10.7717/peerj.2945 (PMC5274519; doi:10.7717/peerj.2945)
Supplement: File S6 [file peerj-05-2945-s006.zip › Supp-File6-metagenome-phylosift-taxonomy-krona-graphs/ESOM-Bin4-Methylococcaceae-phylosift-taxonomy.html]

Javascript must be enabled to view this page.

abundanceMethylo-last-try6-merged-mapped-plus-mates-forward.fastq9214.388325868249214.371147774639142.433435340947925.314825477667818.947811948757452.421864022545493.96414422046662.79441122260596.3007480021237995.519493532004725.749073648485362.874536824243269.770419883516134.885209941758456.121679618516304.081119745678152.0405598728391377.96273732483309.203490614434154.601745307217546.7456787566921056.151177348021056.1511773480295.983244535579595.6617106161805264.713059970913203.277572029242333.880964452658223.556991945147121.672223774088104.53488880592598.5825476008352

  
