# Supplementary material for: Metagenomic identification of active methanogens and methanotrophs in serpentinite springs of the Voltri Massif, Italy
Source: PeerJ. 2017 Jan 26;5:e2945. doi: 10.7717/peerj.2945 (PMC5274519; doi:10.7717/peerj.2945)
Supplement: File S6 [file peerj-05-2945-s006.zip › Supp-File6-metagenome-phylosift-taxonomy-krona-graphs/ESOM-Bin5-Desulfovibrionales-phylosift-taxonomy.html]

Javascript must be enabled to view this page.

abundancemerged-delta-merged-mapped-plus-mates-forward.fastq37367.085129214637367.085129214637139.1443879841872.047605173854524.396053895894374.52041589877133602.6365042045916.522830672014610.284623852615600.91934688044432242.746988223632113.104466662830941.216932530415271.061818447311260.3713940635717.339749356559557.872143425214583.6807426418210673.23860766932064.990429390191376.66028626013688.3301431300641434.27409876852956.182732512344478.091366256172502.977523319711401.701098135856385.940450829314385.624428084118385.308405338921384.992382593724

  
