# Supplementary material for: Metagenomic identification of active methanogens and methanotrophs in serpentinite springs of the Voltri Massif, Italy
Source: PeerJ. 2017 Jan 26;5:e2945. doi: 10.7717/peerj.2945 (PMC5274519; doi:10.7717/peerj.2945)
Supplement: File S6 [file peerj-05-2945-s006.zip › Supp-File6-metagenome-phylosift-taxonomy-krona-graphs/GOR34-river-2012a-metagenome-phylosift-taxonomy.html]

Javascript must be enabled to view this page.

abundanceGORupA1.forward.decontam.derep.adapt\_trim.qual\_trim.fastq.gz145751.025557826145730.943315768137584.6715279334848.814654368743132.1147503152318.3607713084479531.528967883911639.3330302052178.882316418611771.314156359121870.359412266225277.077848315564191.73927312393789.4742945473157752.9972334145003.3647721877320.872506755243390.260855683182604.9901353765930641.40485382466089.110456376241607.57263375742101.839740756373227.289693440891530.625095122584490.614665996793019.607059559752887.094326871391874.627790544221841.354307684915205.887902554235006.975540284873722.207259670183633.213534531483024.418066523362040.060159286821603.5457585958125453.678739341324879.605764790620264.915812280419763.772626789618493.758760832916778.24362843221500.497352942041875.707886367641838.502694757825897.418696525641752.671075879723240.314782450422259.836079137424150.438429678543622.657957251193515.956099710512631.517600753896409.635776783171981.995135371471736.50045715845

  
