# Supplementary material for: Metagenomic identification of active methanogens and methanotrophs in serpentinite springs of the Voltri Massif, Italy
Source: PeerJ. 2017 Jan 26;5:e2945. doi: 10.7717/peerj.2945 (PMC5274519; doi:10.7717/peerj.2945)
Supplement: File S6 [file peerj-05-2945-s006.zip › Supp-File6-metagenome-phylosift-taxonomy-krona-graphs/GOR34-river-2013b-metagenome-phylosift-taxonomy.html]

Javascript must be enabled to view this page.

abundanceGORupB1\_2013.forward.decontam.derep.adapt\_trim.qual\_trim.fastq.gz288085.974126664288064.6296443280306.3039481954172.11516298518186778.05321462231149.852804209719744.884072445312914.10773863256007.642736282593364.63367889482932.60926388451145472.87388389792787.730975383144326.093968321629524.720178162323235.029347569432.023628800684716.011814400347513.314888157053756.657444078523627.629552775273170.1379091894638474.03442435628126.200557695923851.994917314243245.9226274445514839.60029938113124.386952418313090.050481418934583.363640058734194.88246170817014.769793609916771.93187000814782.192006461810278.95573068368401.014548440634200.5072742203116193.927123941916161.57467823453263.392356796417090.9476746564928098.296891333527686.32050257696739.659436448586263.799905153145108.621926096763416.7787898368216523.018385344316453.208627879415214.46866894149464.127411272253468.420234315173079.202537266213701.825778328513313.108511176413217.955676476415555.89448193813

  
