# Supplementary material for: Metagenomic identification of active methanogens and methanotrophs in serpentinite springs of the Voltri Massif, Italy
Source: PeerJ. 2017 Jan 26;5:e2945. doi: 10.7717/peerj.2945 (PMC5274519; doi:10.7717/peerj.2945)
Supplement: File S6 [file peerj-05-2945-s006.zip › Supp-File6-metagenome-phylosift-taxonomy-krona-graphs/GOR34-spring1-2013b-metagenome-phylosift-taxonomy.html]

Javascript must be enabled to view this page.

abundanceGOR1\_CDEF\_2013.forward.decontam.derep.adapt\_trim.qual\_trim.fastq.gz246256.91174094246245.227536387235129.32252723725201.88450344983951.122530190922686.3350928831918401.105916307912847.23485016793598.6106332756141301.3325491328372.473722341928529.36982070179905.89657554619712.6153587287213233.23177076049779.032166714882531.165717028396889.625330166445702.0949126293396329.497529615387017.485344961465231.60075725124024.063166013513282.280426598382751.400941134093252.649194421615085.075173071683390.050115381116037.4168703298018.7084351645114152.62121829378772.472807398812679.603066601782486.87558052833796.830457521373482.3904551736215697.582224945514766.72769813124216.000614710563359.041307685133771.924111571672722.59722796252675.35202894912579.398582792752482.4442155207817362.19522426595409.680652890132711.143538560213977.19165495443629.337703191715041.017439796552800.998076348892981.860735140172846.709364808288747.31064435452

  
