# Supplementary material for: Metagenomic identification of active methanogens and methanotrophs in serpentinite springs of the Voltri Massif, Italy
Source: PeerJ. 2017 Jan 26;5:e2945. doi: 10.7717/peerj.2945 (PMC5274519; doi:10.7717/peerj.2945)
Supplement: File S6 [file peerj-05-2945-s006.zip › Supp-File6-metagenome-phylosift-taxonomy-krona-graphs/GOR34-spring3-2012-metagenome-phylosift-taxonomy.html]

Javascript must be enabled to view this page.

abundanceGOR3AB.forward.decontam.derep.adapt\_trim.qual\_trim.fastq.gz125264.933337104125259.036988556118636.9938358547943.935277972476433.073616419514475.394144359081284.3239816186781633.70913287853162.094521105835278.565425485812523.094562952942459.145233215723960.511091190193567.892974027251572.723777981168699.527612154463420.782439925446859.6593470772870.469429248832057.678168308221264.01135139182119.259989591992346.178547179453771.965309318392514.643539545611257.32176977281311.2328570280411462.74857896995731.3742894849611596.14319150427177.932844218081728.721150654044551.571845586114276.378885553662627.09346376612187.627366989681748.161270213251308.695173436833521.719054331823274.808711939897588.991243423572367.32227308362060.304777017581650.00281573081559.388924497214010.356446258952611.686706443691981.418087311751552.295932140041529.105640092421495.74628014442

  
